# User Participation in Coproduction of Health Innovation: Proposal for a Synergy Project

**DOI:** 10.2196/resprot.9322

**Published:** 2018-05-09

**Authors:** Jens Nygren, Elena Zukauskaite, Niklas Westberg

**Affiliations:** ^1^ School of Health and Welfare Halmstad University Halmstad Sweden

**Keywords:** user participation, coproduction, health, innovation

## Abstract

**Background:**

This project concerns advancing knowledge, methods, and logic for user participation in coproduction of health innovations. Such advancement is vital for several reasons. From a user perspective, participation in coproduction provides an opportunity to gain real influence over goal definition, design, and implementation of health innovations, ensuring that the solution developed solves real problems in right ways. From a societal perspective, it’s a mean to improve the efficiency of health care and the implementation of the Patient Act. As for industry, frameworks and knowledge of coproduction offer tools to operate in a complex sector, with great potential for innovation of services and products.

**Objective:**

The fundamental objective of this project is to advance knowledge and methods of how user participation in the coproduction of health innovations can be applied in order to benefit users, industry, and public sector.

**Methods:**

This project is a synergy project, which means that the objective will be accomplished through collaboration and meta-analysis between three subprojects that address different user groups, apply different strategies to promote human health, and relate to different parts of the health sector. Furthermore, subprojects focus on distinctive stages in the spectrum of innovation, with the objective to generate knowledge of the innovation process as a whole. The project is organized around three work packages related to three challenges—coproduction, positioning, and realization. Each subproject is designed such that it has its own field of study with clearly identified objectives but also targets work packages to contribute to the project as a whole. The work on the work packages will use case methodology for data collection and analysis based on the subprojects as data sources. More concretely, logic of multiple case studies will be applied with each subproject representing a separate case which is similar to each other in its attention to user participation in coproduction, but different regarding, for example, context and target groups. At the synergy level, the framework methodology will be used to handle and analyze the vast amount of information generated within the subprojects.

**Results:**

The project period is from July 1, 2018 to June 30, 2022.

**Conclusions:**

By addressing the objective of this project, we will create new knowledge on how to manage challenges to health innovation associated with the coproduction process, the positioning of solutions, and realization.

## Introduction

Health innovation refers broadly to products, services, organizations, and dissemination of new knowledge that affects people’s ability to maintain and promote their health and well-being as well as prevent ill health [1]. Health as an arena for innovation is connected to the need for structural transformation because of increased demands on health care by medical advances, changes in diagnostic systems, and an aging population [2-4]. An important principle to guide this transformative process involves targeting individual responsibility or cooperation and is expressed in discourses and practices aiming at “person-centered care” [5]. But despite extensive investments to strengthen the role of the patient or care recipient, health innovations seem difficult to implement in established care settings [2,6].

The Swedish Agency for Health Technology Assessment and Assessment of Social Services [7] highlights the prolonged time it takes before research is put into practice and the lack of influence from patients, clients, and practitioners in formulating the aims of the research. In turn, the Swedish Agency for Health and Care Services Analysis [8] points to problems concerning the implementation of the new Patient Act from 2014, which aims to strengthen the patient’s position through participation in their own health care. Moreover, reports point out difficulties in translating the act into practice because of lack of knowledge and concrete tools to fulfill its intentions. As a consequence of these findings, the Swedish Government promotes “applied welfare research” conducted in cooperation with clients or users as well as practitioners to increase the utilization of knowledge for the people affected by the research question [9].

As for industry, the growing and changing health sector is an arena with considerable potential for innovation and the development of new services and products as well as novel business models; however, the health sector is complicated and difficult to navigate [10]. It is a highly regulated sector with complex ethical dimensions to understand and manage [11]. In addition, services and products for the public health sector need to be validated and verified, and this is a costly and time-consuming process [12]. Another obstacle is the Public Procurement Act, which complicates cooperation between public actors and companies. As a result, the industry selling services and products to the public health sector seldom has a direct relationship with the end user, making it difficult to develop user-centered health innovations [13]. In addition to these aforementioned barriers, there are weaknesses in the design and methods for participation currently used in the health sector [14,15]. A common practice is to use user participation in evaluation but not in the development of products and services, which frequently results in an end product that does not correspond to the needs and wishes of the user group.

Research has shown that companies that interact with users of their products for knowledge exchange benefit from reduced uncertainty regarding demand conditions, are more innovative, and more likely to develop products that will succeed in the market [16-18]. This synergy project creates knowledge that can be used to develop future products and services to be offered to a larger market beyond the scope of the synergy project alone. Taking users as a starting point facilitates the customization process, providing opportunities for the users to gain a product that meets their needs while allowing companies to avoid transaction costs related to transferring customers’ requests collected via surveys and other means [19].

**Figure 1:**
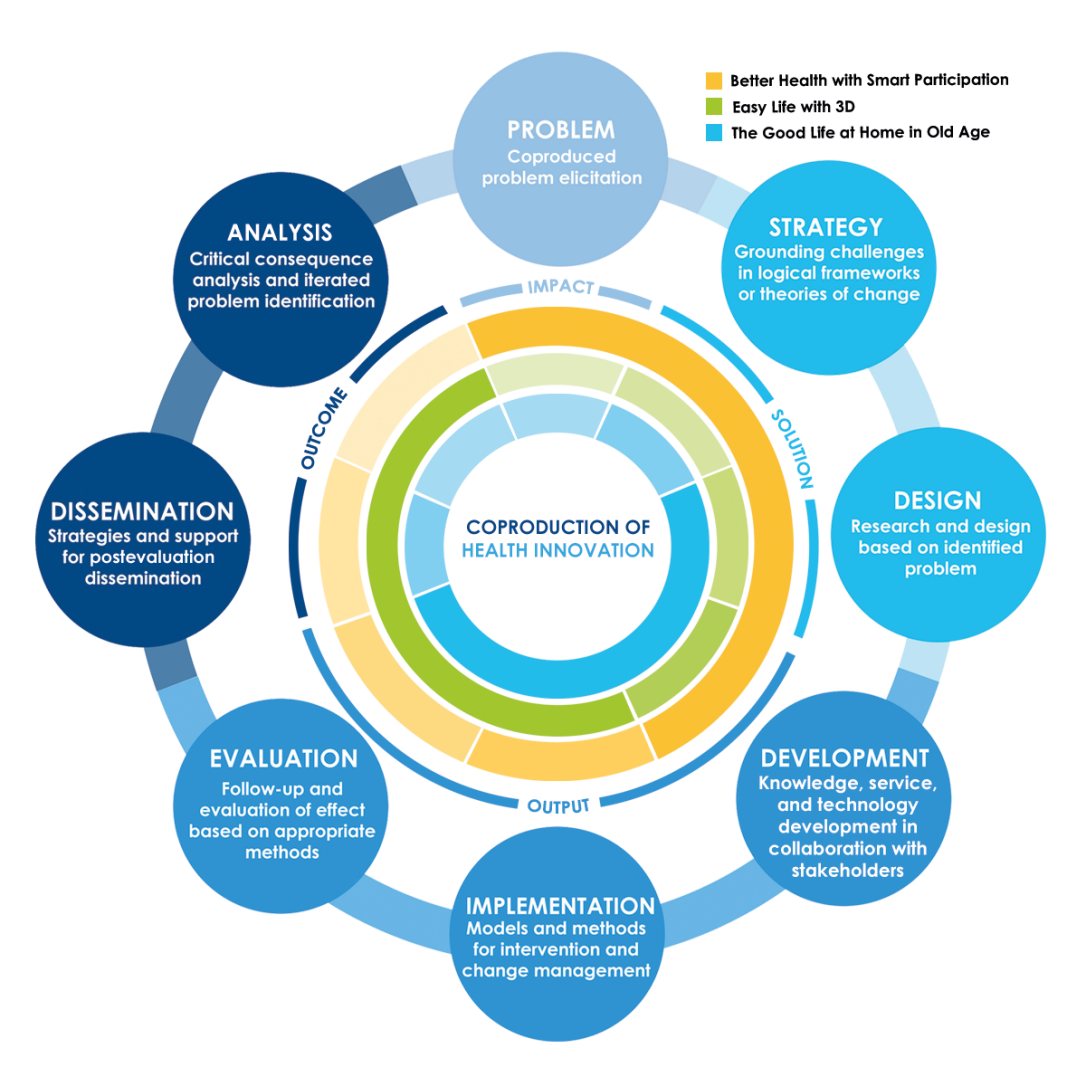
The health innovation process, with 4 stages of value creation—solution, output, outcome, and impact—expressing the logic of change.

Fundamental to this synergy project is that innovation needs to be seen as a process or spectrum ranging from problem elicitation to implementation and dissemination (Figure 1). To meet the described problems and challenges, the synergy project is founded on a model for coproduction where industry and the public sector participate together with users throughout the innovation processes. The purpose of early coproduction with users is to create solutions that are driven by the users and that meet their actual needs. It also creates *one* integrated process where quality assurance, in the form of validation and verification, and implementation is achieved as part of the innovation process—and not afterward as a costly and time-consuming additional process. The main research question of the project is: *How can user participation be applied to overcome barriers in the coproduction of digital health innovations—in the spectrum from problem elicitation to implementation?*

By addressing this, our goal is to create new knowledge of how to manage challenges to health innovation that are associated with the coproduction process, the positioning of solutions, and realization.

### User-Centered Innovation, Coproduction, and Digitalization

Innovation studies, in general, and health innovation literature, in particular, point out different types of innovations, such as new products, services, processes, methods, markets, and sources of supply [4,20,21]. New procedures, health policy innovations, and strategy innovation are also defined as special types of innovation in relation to health [4]. In each of the subprojects, a new digital health innovation is developed. However, the focus of the synergy project is not on the innovation as such (end result), but on the process of coproduction in which health innovations are designed, developed, and disseminated.

There are many different models of innovation, developed for diverse purposes and aiming at various levels of aggregation. A linear—technology-push—model of innovation conceptualizes innovation as a process triggered by accomplishments in research and development that through production and marketing lead to a commercialized product on the market [22]. According to a chain-linked—market-pull—model of innovation, innovation emerges when the potential in the market has been identified and developed through constant feedback between design, test, and market units, which are embedded in research and knowledge environment [23]. More recent models of innovation put even higher emphasis on the interactive nature of the innovation process, not least arguing for the users as important partners for collaboration in innovation [24,25]. The system of innovation approach views innovation as a cumulative process emerging through systemic interactions of the actors (firms, research organizations, governmental authorities, and customers) embedded in a certain institutional setting, delineated by regional, national, or sectorial boundaries [26-28]. In this literature, users can be viewed as sophisticated buyers, active codevelopers, and value setters, contributing to structural change processes [29]. In our project, we view innovation as a circular process with 4 related stages—solution, output, outcome, and impact (Figure 1). The impact stage describes the process of defining a problem together with users and other stakeholders in relation to a challenge that can be addressed through the introduction of a new innovation.

The solution stage describes the design of strategies for how a solution to the defined problem can ground challenges in logical frameworks or theories of change that could be used in the design of solutions together with users and other stakeholders. The output stage describes the process of knowledge, service, and technology development in coproduction with various stakeholders and the implementation of the developed artifact, guided by models and methods for intervention and change management and for evaluation. The outcome stage describes the evaluation of effects of implementation in relation to the defined problem and thereafter dissemination of the results. This includes a critical analysis of the direct and indirect consequences of dissemination and how it contributes toward impact in relation to the initially identified problem. This model, with its 4 stages, has been developed as a part of strategic work to define health innovation efforts at Halmstad University [30]. It goes beyond technology-push or market-pull dichotomy, highlights the iterative nature of innovation, and suggests that innovation can be triggered at any stage of the process. It is inspired by an interactive approach to innovation, emphasizing collaboration with different stakeholders at each stage. In this synergy project, we want to advance our understanding about one type of stakeholders—users; more concretely, we are interested in finding under which circumstances and what roles different groups of users play under different stages of a health innovation process as well as to analyze challenges and mechanisms related to their inclusion. Thus, we are interested in user-centered innovations which we define as triggered by unique user needs and developed in collaboration with product or service providers, public sector, and researchers. Including users and other stakeholders of health products and services in the design and development process leads to increased efficiency and quality of health care processes [31,32].

Various concepts, such as cocreation, codesign, and coproduction, are used to describe innovation processes with several stakeholders involved. Sometimes they are used as synonyms, and sometimes differences are pointed out [33]. Cocreation is the broadest term, including different forms of interaction with users (or other stakeholders) from consultation to participation and generation [34]. Codesign refers to the “creativity of designers and people not trained in design working together in the development process” and focuses on the early stages of the innovation process from idea generation to product development and less so on implementation, dissemination, and evaluation [33]. In this project, we follow Dunston et al’s (2009) understanding of *coproduction* in health, which implies that health innovation evolves in a collaboration process between users, health care professionals, and other stakeholders, such as private companies bringing products and services to the market and researchers at the University [32]. This concept is chosen because it is best aligned with the understanding of the innovation process that is used in this project. It also delineates interaction with users as an active collaboration rather than opening up for any type of activities where users influence innovation processes.

According to health care and innovation scholars, one of the biggest potentials for renewal of the health sector is via *digitalization*, which also has considerable potential to result in cost savings [2,35]. In the Swedish context, it is estimated that digitalization of health care could save 180 billion SEK a year [36]. Furthermore, the use of digital technologies could also lead to better disease prevention, greater personal engagement in one’s health, and an improved work environment for health care professionals [36]. Thus, because of its high innovation and value-added potential, we focus on digital, user-centered health innovations. In this project, we understand digitalization as the use of a variety of converging digital tools (wireless sensors, information systems, social networking, and mobile connectivity) in health-related products and services (see also [2]).

### Subprojects

The synergy project consists of 3 subprojects, allowing us to study user participation and coproduction in different organizational contexts and in relation to different user groups and, thus, to address coproduction-related challenges from different perspectives. Although the projects have a similar emphasis on user participation in coproduction, they also target distinctive phases or segments in the spectrum from problem elicitation to the implementation of health innovation (Figure 1). Implementing these projects in parallel will help gain an in-depth understanding of the challenges and barriers associated with user-centered coproduction and how these barriers can be overcome or managed during the innovation process. The projects are similar in that they are addressing complex health challenges and are designed so that users and other stakeholders have significant influence over the content of the projects. Collectively, our proposed projects are likely to yield new knowledge of these challenges and how to tackle them from a holistic perspective, rather than from a segmented process of innovation.

The project is generic in its ambition to create a meta-framework for solutions to problems that currently exist in the health sector, which transcend the boundaries of each subproject. By studying similarities and differences in our chosen areas of innovation—such as different social and organizational contexts and the needs, abilities, and conditions of different user groups—we will generate knowledge and solutions to creating health innovations that can be applied across a variety of contexts. The research question is multidisciplinary and needs an integrated effort from competencies belonging to different research fields to be answered. Moreover, the project aims at mobilizing researchers and representatives from industry, the public sector, and users in project teams, conducting health innovation in an integrated participatory design process where all actors are involved in the process at all stages.

#### Project 1: Better Health With Smart Participation

Digital communication tools through coproduction with children in habilitation target challenges in coproduction where children participate in the development of digital health, promoting services in relation to their own health. The context for application of such services is, in this case, health care and habilitation for children with disabilities. In relation to our model for innovation (Figure 1), the project specifically aims to strengthen coproduction with children in problem elicitation, design, development, and implementation.

#### Project 2: Easy Life With 3D

Personalized assistive devices through coproduction with people with functional disabilities address our identified challenges by applying coproduction in digital visualization technology and rapid prototyping, at the Fab Lab facility at Halmstad University. The products coproduced target people with functional disabilities and are primarily concerned with the development, implementation, evaluation, and dissemination stages in the innovation model (Figure 1).

#### Project 3: The GoodLlife at Home in Old Age

Intelligent age–friendly homes through coproduction with elderly people address the research question by providing an understanding of challenges for coproduction in the contexts of people’s own home environment and in relation to home care. The target group is the older people, and the aim of the project is to coproduce innovative solutions to strengthen the possibility to remain at home and gravitates mainly around the steps of implementation, evaluation, and dissemination in our model for innovation (Figure 1).

### Work Packages

Our rationale for this synergy is that the integration of users in the coproduction of health innovations contributes to relevance and quality of innovations as well as to efficiency in the innovation process. However, achieving such user participation can be challenging both in itself and because of circumstances and practices associated with various contextual factors. We have specifically identified 3 areas of challenges, namely, *coproduction*, *positioning*, and *realization*. These challenges are influenced by factors associated with different contexts and user groups, and therefore need to be studied from a variety of perspectives to create a broad understanding of the meaning of the challenges and how they can be managed at a general level. We intend to accomplish this by integrating 3 subprojects that focus on different user groups, apply different strategies to promote human health, and relate to distinct parts of the health sector.

#### Work Package 1—Coproduction

The coproduction work package targets how problem formulation and development of a logical rationale for health innovation is in need of early collaboration with users. It also deals with barriers to designing health innovations with vulnerable groups. The work package is divided into 3 parts:

- *Method for early collaboration with users:* Design a method for early user involvement in problem elicitation from which a rationale and logic for health innovation can emanate.
- *Framework describing barriers:* Identify barriers to working with vulnerable groups to bridge the gap between users, industry, health professionals, and researchers.
- *Conditions for coproduction:* Pinpoint problems relating to questions of ownership, ethics, and legal matters in the coproduction of products and services in the health sector, including experiences of the view of users, industry and public sector representatives, and researchers.

#### Work Package 2—Positioning

The positioning work package is focused on the strategic adaptation of health innovation in relation to laws and regulation in the health sector, existing organizational contexts, and routines to prepare for future implementation. It draws on the experiences from the 3 subprojects to develop generic strategies for how and when to introduce health innovations based on user needs and perspectives as well as contextual factors relevant to other stakeholders:

- *Regulatory framework:* Create a framework for prospective adaption of health innovations to fit laws, regulations, and ethical dimensions in the health sector to facilitate future implementation.
- *Planning model for implementation:* Create a method-driven and generic model for how to work with the prospective adoption of health innovations to tackle barriers for implementation related to organizational contexts, routines, hierarchy, and inertia in systems.
- *Patterns of onboarding:* Develop a methodology that can be used generically to establish where in an existing ethical, regulatory, and organizational framework health innovations have potential to be introduced and identify actors that play a key role in facilitating service onboarding and use.

#### Work Package 3—Realization

The realization work package aims to frame strategies for successful implementation, evaluation, and dissemination of health innovations that are coproduced with users:

- *Preservation of knowledge:* Establish design tools that convey the knowledge, values, and qualities originating from user participation in coproduction to be integrated into new solutions and to make sure that this information is preserved and continues to convey users’ perspectives in future refinement or development of the innovation.
- *Framework evaluating implementation:* Use existing models for process and impact evaluation of implemented health promotion interventions as the basis for developing a broad evaluation framework that takes into account user perspective in evaluating implementation.
- *User-centered business models:* Increase knowledge on how to combine profitability and user value into viable business models for coproduced health innovations and how these issues can be dealt with already at the problem elicitation and design phases of the innovation process.

## Methods

### Overview

The synergy subprojects are designed in such a way that each of them can be carried out independently of the others. The subprojects use a variety of methods to generate empirical data adapted to circumstances and conditions for the individual project, and to the different phases of the innovation process (Figure 1). In identifying challenges and problems for the involved user groups, individual and focus group interviews will be conducted, as well as observation of users in the context of their everyday life. In the phase of creation of health innovations, the workshop format plays a significant part, and using a mixture of methods—storyboards, scenarios, analogies, etc—user groups, researchers, and company representatives work together iteratively to find solutions to identified problems. To evaluate outcomes of implementation and interventions, both quantitative and qualitative methods are used, in the format of validated questionnaires as well as individual and focus group interviews.

### Data Collection and Analysis

The work on the work packages will use a case methodology for data collection and analysis based on the subprojects as data sources. Each subproject will be treated as a study object where documentation and results from the project will be used as data for qualitative analysis and design of frameworks and models that answer the goals described in the respective work packages [17,37]. A case study approach is beneficial in several ways for this project because it allows for a combination of data collection tools, gives attention to contextual factors regarding the phenomena under investigation, and can be used to develop theory [38]. Furthermore, in our ambition to develop a generic, as well as context-sensitive, understanding, the use of a multiple case study logic is vital. Our chosen cases (the subprojects) are similar in its attention to user participation in coproduction, but unalike regarding, eg, context and target groups. This allows for an examination of similarities and dissimilarities, patterns, and particulars in relation to each of the challenges (coproduction, positioning, and realization elaborated on in WP 1-3) and as such develops a generalized as well as context-sensitive understanding of user participation. The analysis of the research question at the synergy level will use strategies developed using the framework methodology to handle and analyze the vast amount of information generated within the subprojects [39]. This method uses matrices to summarize, compare, and synthesize diverse units for analysis.

### Dissemination of Results

Coproduction in the health sector is complex, and the prerequisites and opportunities for coproduction are dependent on the target group, the type of innovation being developed, and the target health sector. Therefore, lessons learned from the respective subprojects will be shared to maximize the success of each project as well as to the overall synergy project. The results presented in the work packages will strengthen the ability for coproduction within the involved research groups, companies, public sector, and user associations, and this acquired expertise will, through established networks and collaborations, be disseminated to other researchers within and outside the University as well as to partners and the involved stakeholders. The work packages have 2 types of deliverables. *Reports* are small publications or presentations aimed at (1) spreading experiences, methods, and new knowledge between the 3 subprojects and the participating partners from industry and the public sector early in the course of the project and (2) making sure that the knowledge generated in the project remains with the project partners beyond individuals. For some work packages, preliminary reports will be released early internally and then completed as final reports for dissemination outside the synergy project group, such as to interested recipients within industry, public sector, and nongovernmental organizations. Such dissemination can be in the forms of presentations, popular science articles, information booklets, news articles to be spread via conferences, industry breakfast meetings, industry magazines, specialized websites, social media, and other relevant mediums. In addition to researchers, representatives from companies, public sector, and user organizations will be involved in these dissemination tasks. *Papers* are in the format of in-depth research publications based on the various reports and are developed to meet criteria for scientific evidence.

### Project Organization

The synergy project team will be responsible for coordinating and addressing the main research question with its work packages, but collaboration with companies and users will be paramount at this level, and not only in the subprojects. This collaboration at the synergy level will be organized in the format of workshops involving researchers, companies, and users. At these workshops, preliminary results and findings will be discussed and elaborated and function as a springboard to achieve deliverables in the form of reports and papers. An essential objective of these workshops is to identify novel knowledge that constitutes intellectual assets, such as scientific results, methods, and inventions, and how to use them for various purposes. To manage questions of ownership and availability, the Intellectual Asset Inventory will be used (Chalmers Innovation office [40]).

## Results

The project period is from July 1, 2018, to June 30, 2022. Outputs from the work packages will be reported continuously throughout the project period, such as internal reports, films, posters, exhibitions, prototypes, press releases, and finally as research publications.

## Discussion

In this synergy project, we want to advance our understanding about one type of stakeholders—users; more concretely, we are interested in under which circumstances and what roles different groups of users play under different stages of a health innovation process as well as to analyze challenges and mechanisms related to their inclusion. This project contributes to the literature on health innovation in several ways. First, it provides an in-depth understanding on how user participation, and mechanisms for their inclusion, changes as the innovation process evolves, whereas most previous studies focus either on early stages of the process or verification. Second, the project places the user and the innovation process in a context, addressing the issues related to legal, ethical, and organizational aspects of the environment of coproduction processes, rather than focusing only on the added value of users’ knowledge and ways to get access to it. Third, in this project, we focus on the coproduction with vulnerable groups of users such as children, older people, and people with disabilities. Previous research has shown the capability of these groups in participating in innovation processes as well as the added value of such participation [14,33,41]. This project will contribute to the research field by explicitly discussing mechanisms and challenges related to their inclusion.
